# Integrated metabolomic and transcriptomic analyses provide insights into regulation mechanisms during bulbous stem development in the Chinese medicinal herb plant, *Stephania kwangsiensis*

**DOI:** 10.1186/s12870-024-04956-2

**Published:** 2024-04-11

**Authors:** Hao Huang, Ying Wei, Shaojun Huang, Shijian Lu, Huasheng Su, Liuhui Ma, Weiping Huang

**Affiliations:** 1 Guangxi Vocational University of Agriculture, Nanning, 530009 China; 2 Guangxi Botanical Garden of Medicinal Plants, Nanning, 530023 China

**Keywords:** Stephania kwangsiensis, Bulbous stem, Development, Metabolomic analysis, Transcriptomic analysis

## Abstract

**Background:**

*Stephania kwangsiensis* Lo (Menispermaceae) is a well-known Chinese herbal medicine, and its bulbous stems are used medicinally. The storage stem of *S. kwangsiensis* originated from the hypocotyls. To date, there are no reports on the growth and development of *S. kwangsiensis* storage stems.

**Results:**

The bulbous stem of *S. kwangsiensis*, the starch diameter was larger at the stable expanding stage (S3T) than at the unexpanded stage (S1T) or the rapidly expanding stage (S2T) at the three different time points. We used ultra-performance liquid chromatography-tandem mass spectrometry (UPLC-MS/MS) and Illumina sequencing to identify key genes involved in bulbous stem development. A large number of differentially accumulated metabolites (DAMs) and differentially expressed genes (DEGs) were identified. Based on the differential expression profiles of the metabolites, alkaloids, lipids, and phenolic acids were the top three differentially expressed classes. Compared with S2T, significant changes in plant signal transduction and isoquinoline alkaloid biosynthesis pathways occurred at both the transcriptional and metabolic levels in S1T. In S2T compared with S3T, several metabolites involved in tyrosine metabolism were decreased. Temporal analysis of S1T to S3T indicated the downregulation of phenylpropanoid biosynthesis, including lignin biosynthesis. The annotation of key pathways showed an up-down trend for genes and metabolites involved in isoquinoline alkaloid biosynthesis, whereas phenylpropanoid biosynthesis was not completely consistent.

**Conclusions:**

Downregulation of the phenylpropanoid biosynthesis pathway may be the result of carbon flow into alkaloid synthesis and storage of lipids and starch during the development of *S. kwangsiensis* bulbous stems. A decrease in the number of metabolites involved in tyrosine metabolism may also lead to a decrease in the upstream substrates of phenylpropane biosynthesis. Downregulation of lignin synthesis during phenylpropanoid biosynthesis may loosen restrictions on bulbous stem expansion. This study provides the first comprehensive analysis of the metabolome and transcriptome profiles of *S. kwangsiensis* bulbous stems. These data provide guidance for the cultivation, breeding, and harvesting of *S. kwangsiensis.*

**Supplementary Information:**

The online version contains supplementary material available at 10.1186/s12870-024-04956-2.

## Introduction

*Stephania kwangsiensis* Lo (Menispermaceae), a well-known Chinese herbal medicine, is an endangered plant distributed from northwestern to southwestern Guangxi Province and southeastern Yunnan Province of China. Specific secondary metabolites of Chinese herbs such as phenylpropanoids and alkaloids exert pharmacological effects. Some natural products share common upstream pathways and contain a limited number of common precursors [1–3]. The main medicinal parts of *Stephania kwangsiensis* are spheroids or depressed globose tuberous roots rich in alkaloids. The tuberous roots of *Stephania kwangsiensis* are used to treat pain in the stomach, duodenal ulcers, pyrexia, upper respiratory tract infection, acute gastroenteritis, toothache, dysentery, neuralgia, bruising, and swelling [1, 2]. The main alkaloids present in the root tubers of *Stephania kwangsiensis* include *L*-roemerine, dehydroroemerine, *D*-isocorydine, corydine, *L*-tetrahadropalmatine, palmatine, palmatine chloroform, sinoacutine, stephanine, dehydeostephanine, and *L*-capaurine, among others [1]. The *L*-tetrahydropalmatine (craniodyne, also known as rotundine) content was more than 2% [4]. It can relieve pain and fever, reduce the drug demand threshold, and drug dependence, and inhibit colorectal cancer [5, 6]. Sinoacutine from *S. kwangsiensis* can improve the pain threshold by electrical stimulation of the toes on hot plates [7]. However, little is known about the dynamic changes in secondary metabolites that are important for harvesting high-quality roots of *S. kwangsiensis*.

In addition, *S. kwangsiensis* seeds are difficult to germinate under natural conditions because of the hardness of the seed shell. The slow growth of roots, indiscriminate digging, and destruction of suitable wild environments by human activities have led to a decrease in the annual population of this species [1]. Therefore, the breeding and cultivation of *S. kwangsiensis* are highly important.

Currently, relevant studies on the geophytes of *S. kwangsiensis* have predominantly focused on the analysis of alkaloid components in root tubers, antibacterial effects of endophytic fungi on plants and animals, application of extracts in biological control, biogenetic diversity, separation and extraction of active components, and tissue culture [1]. To date, there have been no reports on the growth and development of *S. kwangsiensis*, and the molecular mechanisms affecting the expansion of bulbous stems and accumulation of medicinal components during the growth and development of *S. kwangsiensis* are unknown.

In the early germplasm collection, preservation, and breeding process, we found that the main medicinal part of *S. kwangsiensis* is the bulbous stem, which developed from the hypocotyl rather than the root. This type of bulbous stem is also known as a swollen hypocotyl and is a type of underground plant stem. The enlarged underground stem of sugar beet (*Beta vulgaris*) originates only from the hypocotyl. The hypocotyl of some plants expands with the stem or adjacent parts of the root system, such as the enlarged stem of turnips (*Brassica spp.*) or the swollen root system of Adenia [8]. The underground storage organ of a crop beet is also known as the storage root (taproot), which originates from the hypocotyl and primary roots [9]. Taproot formation is the result of the rapid growth of secondary xylem in the hypocotyl [10]. Multiple plant hormones, including auxins and gibberellins (GA), are important for taproot enlargement [10–15]. Plant hormones and sugars affect turnip taproot initiation and development [11]. Starch and sucrose metabolic pathways are altered during turnip taproot development [16]. In addition, reduced lignification and altered cell wall metabolism have been suggested to contribute to the loosening and elongation of the cell wall, thus promoting the enlargement of taproots [14, 17].

This study used ultra-performance liquid chromatography-tandem mass spectrometry (UPLC-MS/MS) and Illumina RNA-seq to perform metabolome and transcriptome profiling of *S. kwangsiensis* bulbous stems at three different developmental stages. This study provides insights into the molecular mechanism of *S. kwangsiensis* bulbous stem growth and development and provides a theoretical basis for the cultivation, breeding, and harvesting of *S. kwangsiensis.*

## Results

### Different growth periods in *S. kwangsiensis*

**Fig. 1:**
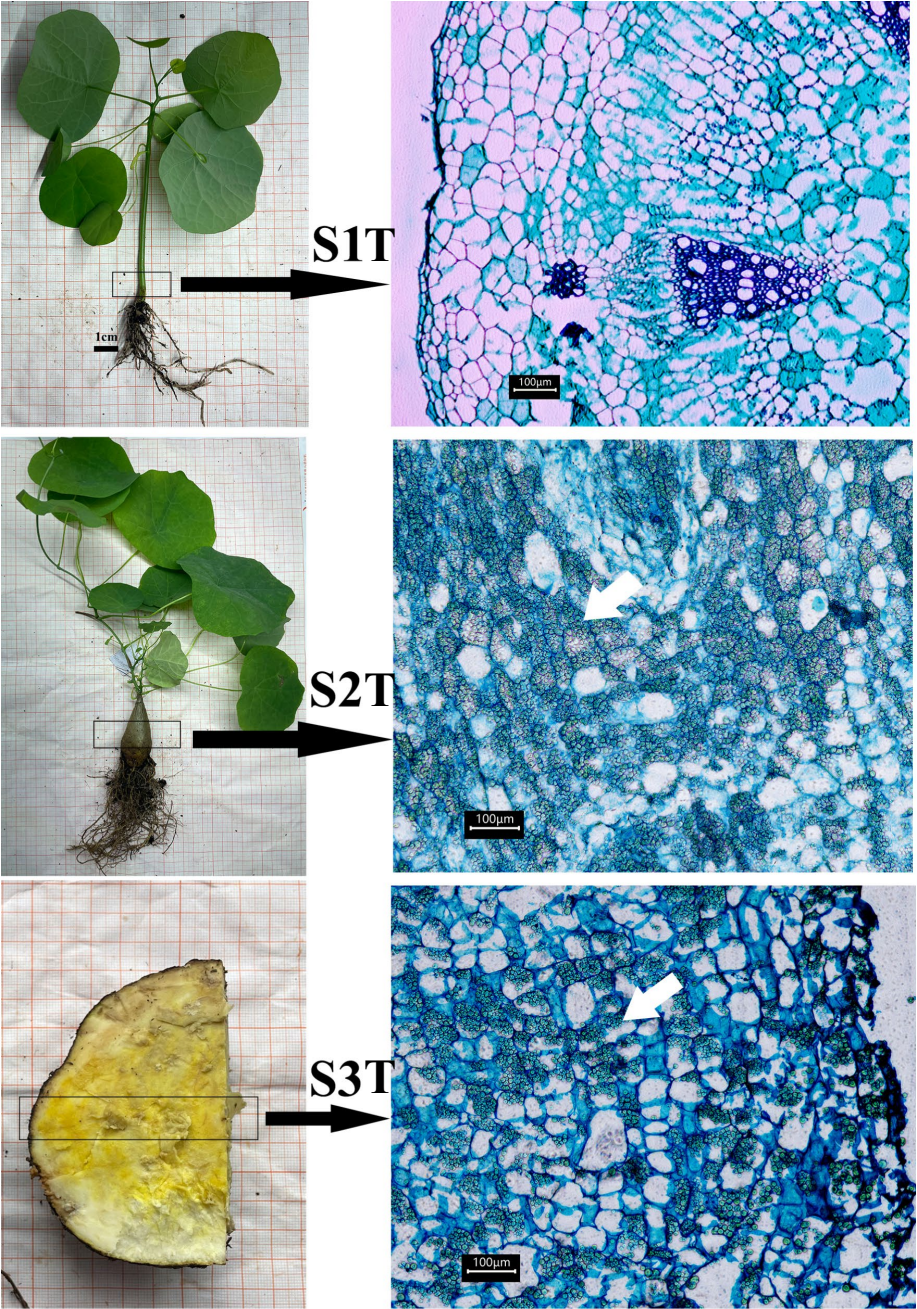
Different growth periods and slice analysis of Stephania kwangsiensis. The cell micrograph on the right shows the black box on the left

Several phenotypes of *S. kwangsiensi*s occur at various growth stages, with the bulbous stem being the primary therapeutic component (Fig. 1). The stem did not expand during the unexpanded stage (S1T), and the cell shape was relatively regular and polygonal, with thin cell walls and almost no intercellular gaps. The size of the cells varied and almost no starch granules were present (Fig. 1). In the rapidly expanding stage (S2T), the focus was on storing thin-walled tissue; the cell wall was thick, and the shape of the cells was irregular. Many small starch particles were formed within the cells, and most were evenly distributed within the cells. The number of starch particles significantly increased; however, the number of large starch particles remained relatively low (Fig. 1). In the stable expansion stage (S3T), the focus is on storing thin-walled tissues with thickened cell walls and larger cell gaps. Starch particles were concentrated in one corner of the cell and the number of large starch particles gradually increased (Fig. 1).

### Metabolomic changes associated with the growth period

Significantly different phenotypes at different periods suggest differential concentrations of metabolites in *S. kwangsiensis*. Therefore, we performed a metabolome analysis using UPLC-MS/MS to obtain the metabolome profile of *S. kwangsiensis* (Fig. S1). We detected 1,068 metabolites, that were classified into 10 classes: 216 alkaloids, 161 lipids, 132 phenolic acids, 128 amino acids and derivatives, 114 others, 102 flavonoids, 83 organic acids, 69 nucleotides and derivatives, 34 terpenoids, 29 lignans, and coumarins (Table S1, Fig. S2). These results suggest that alkaloids, lipids, and phenolic acids are the main metabolites of *S. kwangsiensis* during the growth period.

To evaluate the metabolome differences between groups and the variation status among the three replicates, we performed principal component analysis (PCA) of all samples. The PCA plot showed that the samples were clearly distinguished between the three growth periods, indicating substantial differences in metabolite concentrations in *S. kwangsiensis* (Figs. S3-S4). The replicates clustered together in the PCA, suggesting low variability in the metabolome profile (Fig. S5A). The Pearson’s correlation coefficient was used to test for correlations between different samples. The results also showed that the duplicate samples exhibited a strong correlation (Fig. S5B).

**Fig. 2:**
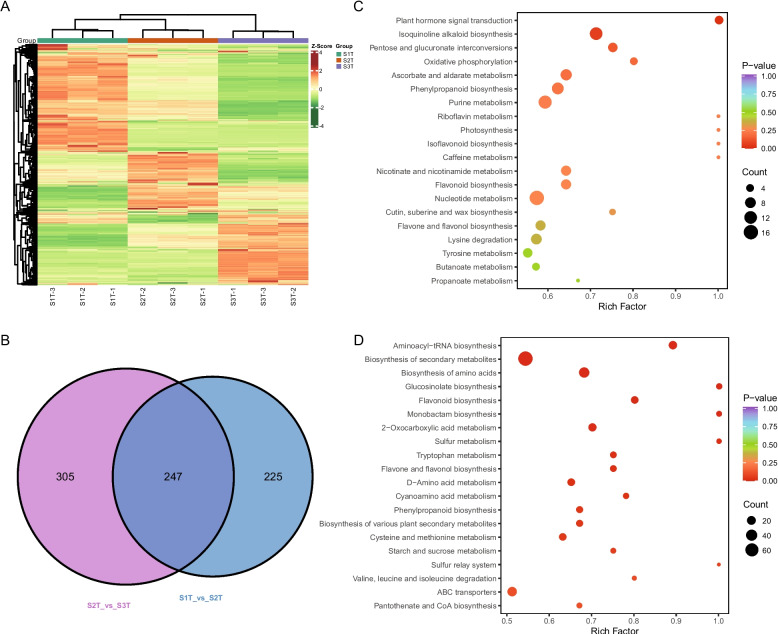
Metabolomic analysis of the growth period of *S. kwangsiensis*. Heatmap of differentially accumulated metabolites in each sample (**A**); number of differentially accumulated metabolites (**B**); pathway enrichment analysis for differentially abundant metabolites: S2T-vs-S3T (**C**); S1T-vs-S2T (**D**)

The heatmap illustrates nine samples divided into three main clusters based on ion abundance. The samples from each period were aggregated (Fig. 2A). Accordingly, more metabolites in *S. kwangsiensis* were abundant in S3T than in S1T or S2T (Fig. 2A). To explore metabolite differences in *S. kwangsiensis* during the three periods, we compared the abundance of metabolites.

The abundance of lipids in S1T was lower than those in S2T and S3T. Among the S3T alkaloids, lipids, and phenolic acids were the top three differential classes based on their abundance (Table S2; Fig. S2). We obtained 472 differentially accumulated metabolites (DAMs) in the comparison between S1T and S2T and 552 DAMs in the comparison between S2T and S3T, with threshold values of variable importance in projection (VIP) score ≥ 1 and fold change ≥ 2 (Fig. 2B). A total of 247 DAMs were common, suggesting variation in the DAMs present during different growth periods (Fig. 2B). We used a differential abundance score to detect global changes in metabolites based on the Kyoto Encyclopedia of Genes and Genomes (KEGG) pathway enrichment analysis. Plant hormone signal transduction, isoquinoline alkaloid biosynthesis, and pentose and glucuronate interconversion pathways were significantly enriched, with multiple upregulated DAMs in S2T compared to S3T (Fig. 2C). Aminoacyl-tRNA biosynthesis, biosynthesis of secondary metabolites, and biosynthesis of amino acids were significantly enriched by multiple upregulated DAMs in S1T compared to S2T (Fig. 2D).

Overall, these results indicated that with plant growth, there were significant changes in the pathways mainly related to growth in the early stages and secondary metabolism in the later stages.

### Global transcriptomic changes in different growth periods of *S. kwangsiensis*

**Fig. 3:**
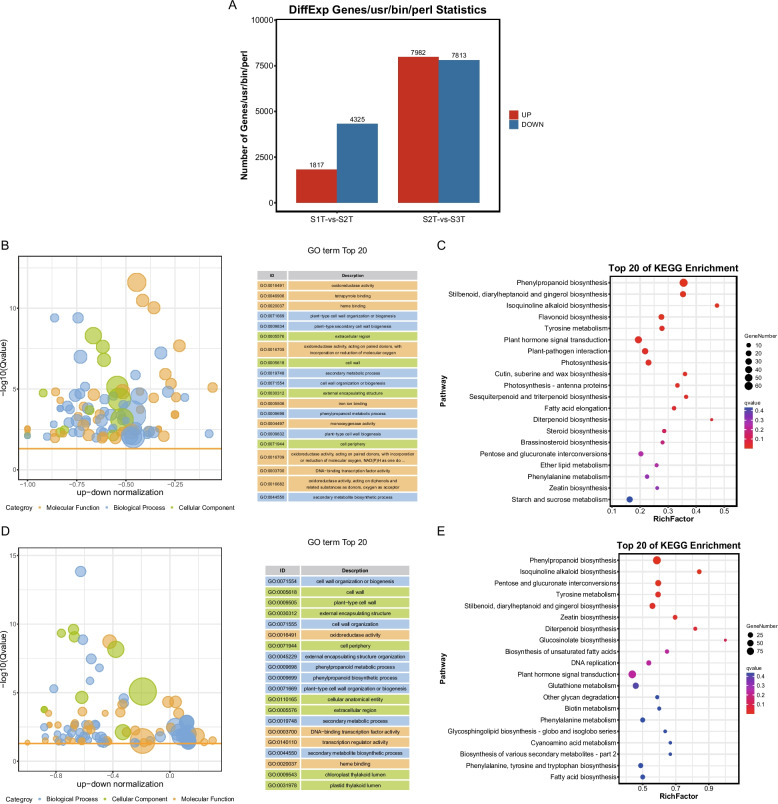
Gene expression analysis. Number of differentially expressed genes between different groups (**A**); GO enrichment analysis of the differentially expressed genes (DEGs) identified in S1T-vs-S2T (**B**); KEGG pathway enrichment analysis of DEGs identified in S1T-vs-S2T (**C**); GO enrichment analysis of DEGs identified in S2T-vs-S3T (**D**); KEGG pathway enrichment analysis of the DEG identified in S2T-vs-S3T (**E**)

RNA was extracted from the samples used for metabolomic analysis and analyzed using RNA-seq. The number of raw reads among the samples ranged between 67,344,314 and 92, 093, 962, respectively. After removing low-quality reads, the Q30 scores of all products were greater than 93%, indicating high-quality gene sequencing results for downstream analysis (Table S3). A total of 63,851 assembled unigenes with an average length of 1001 bp were aligned to multiple databases, including KEGG, NCBI non-redundant (NR), Swiss-Prot, Gene Ontology, and Clusters of Orthologous Groups/EuKaryotic Orthologous Groups (COG/KOG), to annotate the function of the unigenes, of which 24,541 unigenes were mapped in at least one database (Table S3). Pearson’s correlation coefficient between replicates ranged from 0.999 to 1, suggesting that the transcriptome results were reliable and stable (Fig. S6A). The replicates clustered together in PCA, suggesting low variability in the unigene profile (Fig. S6B). A total of 6,196 differentially expressed genes (DEGs) in the comparison between S1T and S2T and 15,795 DEGs in the comparison between S2T and S3T samples were identified using threshold values of absolute log2 fold change (FC) ≥ 1 and adjusted *P*-value < 0.05, respectively (Fig. 3A, Tables S4 and S5). The expression of key genes was validated by qRT-PCR (Fig. S7), which showed high consistency between the transcriptome and qRT-PCR testing.

To systematically explore the biological functions of DEGs potentially involved in *S. kwangsiensis* at different growth periods, we used DEGs generated by pairwise comparisons of different groups for KEGG pathway enrichment analysis (Fig. 3B, D). Phenylpropanoid biosynthesis, stilbenoid, diarylheptanoid, gingerol biosynthesis, isoquinoline alkaloid biosynthesis, and tyrosine metabolism were significantly enriched in DEGs between the two comparison groups (Fig. 3C, E). Flavonoid biosynthesis and plant hormone signal transduction were identified using KEGG analysis of S1T and S2T (Fig. 3C), and pentose and glucuronate interconversions and zeatin biosynthesis were identified by analyzing S2T and S3T (Fig. 3E). These results are similar to those of the metabolomics.

### Temporal analysis for DEGs across different growth periods of *S. kwangsiensis*

**Fig. 4:**
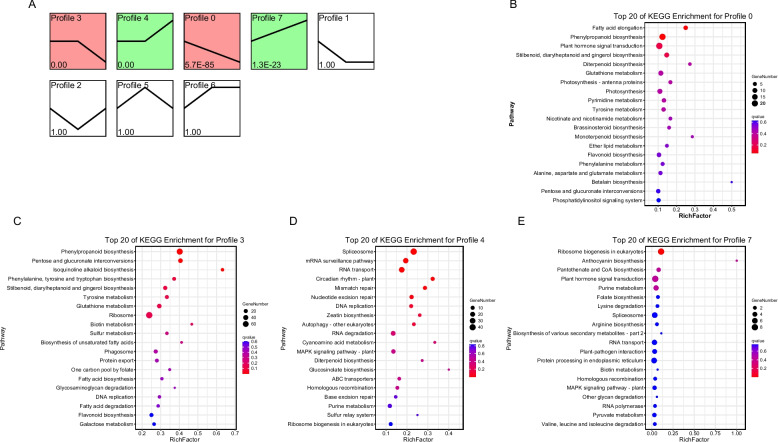
The number of differential clustered genes in different growth periods (**A**); KEGG pathway enrichment analysis for genes in trend profiles that of significant differences: Profile 0 (**B**), Profile 3 (**C**), Profile 4 (**D**), Profile 7 (**E**)

To assess the gene expression patterns during development, the DEGs were separately clustered into eight clusters using the STEM algorithm. The four clusters in each group, including profiles 0, 3, 4, and 7, were significantly enriched by genes based on a* P* ≤ 0.05 (Fig. 4A). For example, the gene levels in profile 7 of both groups showed a rapid increase from S1T to S3T, and those in Profile 4 displayed an increase from S2T to S3T (Fig. 4A). The gene expression levels in profile 0 showed a continuous decrease, whereas those in profile 3 showed a downward trend from S2T to S3T (Fig. 4A). These results indicated that the genes involved in the significant profiles play an important role in the developmental process. Overall, nine KEGG pathways were associated with all profiles. The phenylpropanoid biosynthesis (ko00940) pathway was a common pathway enriched in profiles 0 and 3, and fatty acid elongation (ko00062) belonged to profile 0 (Fig. 4B, C). Pentose and glucuronate interconversions (ko00040) and isoquinoline alkaloid biosynthesis (ko00950) were unique to profile 0 (Fig. 4B). Spliceosome (ko03040), mRNA surveillance pathway (ko03015), RNA transport (ko03013) and circadian rhythm-plant (ko04712) belonging to Profile 4 (Fig. 4D). Finally, only one pathway belonged to profile 7, ribosome biogenesis in eukaryotes (ko00940) (Fig. 4E). Together, these results show that isoquinoline alkaloid biosynthesis and phenylpropanoid biosynthesis play significant roles in developmental processes.

### Key pathways involved in different growth periods of *S. kwangsiensis*

**Fig. 5:**
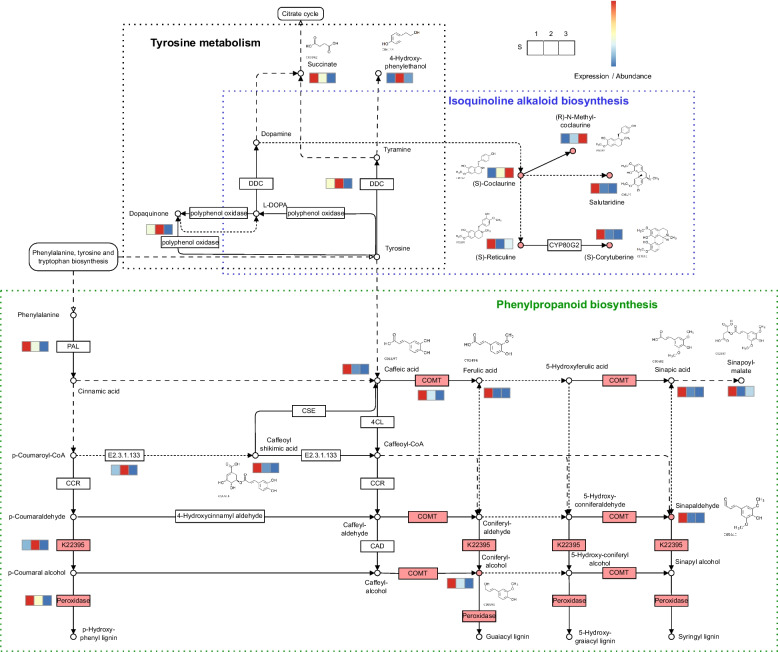
DEGs and DAMs present in the key pathways. The circle with red background represents the key substances with significant difference, while the box with red background represents the key enzymes with significant difference

KEGG analysis was used to annotate the upregulated or downregulated genes and metabolites involved in the biosynthesis of isoquinoline alkaloids biosynthesis and phenylpropanoid biosynthesis (Fig. 5). The number of genes and metabolites with similar regulatory patterns was significantly higher than the number of altered genes and metabolites (*P* = 0.0001, chi-square test). Our non-targeted metabolome analysis indicated significant reductions in salutaridine, (S)-reticuline, (S)-corytuberine, coniferyl alcohol, and sinapaldehyde levels in the S3T group. The accumulation of (S)-coclaurine and (R)N-methyl-coclaurine gradually increased and peaked at S3T. The gene expression patterns of the DEGs in the three periods were consistent with those of the metabolites. The levels of caffeic acid 3-O-methyltransferase (COMT, K13066), cinnamyl alcohol dehydrogenase (K22395), and peroxidase (K00430) were significantly higher in S1T than in S3T. We conducted a correlation analysis of the DEGs and DAMs in key pathways, and most showed significant correlations (Fig. S8). These results suggest that DAMs and DEGs exhibit various regulatory trends depending on the developmental period.

## Discussion

The phenotype of *S. kwangsiensis* changed considerably during all the three growth periods. The sliced cells of *S. kwangsiensis* also showed pronounced changes, especially in the starch granules. The metabolomic results showed that lipids were upregulated during development. Lipids participate in diverse biological functions, including carbon storage [18]. The enlarged bulbous stem is a nutrient storage organ. A variety of nutrients, especially sucrose and starch, are accumulated in taproot originated from the hypocotyl [11, 19]. In addition, the starch and sucrose metabolism pathway is one of the most active pathways in taproot development of turnip and is considered very important for the secondary thickening process of taproots [16]. The enlarged starch granules and increased lipid content indicated that carbon storage increased during bulbous stem development, and these changes may be important for hypocotyl-originated bulbous stem enlargement.

Alkaloids and phenolic acids are the main secondary metabolites produced during the development of *S. kwangsiensis* bulbous stems. Phenolic acids belong to the class of phenylpropanoid metabolites. Secondary metabolites, detected in smaller numbers than alkaloids and phenolic acids, were flavonoids, which are phenylpropyl derivatives. Some of these metabolites have medicinal value and may share a common upstream pathway to form a limited number of common precursors [3, 20]. These data provide information for further studies on the medicinal value and mechanisms of action of *S. kwangsiensis*.

The alkaloid contents of S2T and S3T were higher than that of S1T. *L*-tetrahydropalmatine is an isoquinoline alkaloid with medicinal properties. Although the two end metabolites, (S) -corytuberine and salutaridine, were downregulated in the pathway (Fig. 5), the accumulation of the upstream metabolite, (S)-coclaurine, may have led to an increase in the total alkaloid content. In the KEGG analysis of metabolites, multiple DAMs involved in isoquinoline alkaloid biosynthesis increased significantly from S1T to S2T, indicating that S1T to S2T was the stage of isoquinoline increase, and the medicinal value significantly increased in *S. kwangsiensis*. At the transcriptome level, the expression levels of multiple pathway genes were significantly altered. The phenylpropanoid biosynthesis pathway tended to be downregulated during bulbous stem development. Based on genome-wide DEGs analysis, Li et al. [18] reported that, in addition to the starch and sucrose metabolism pathways, the phenylpropanoid biosynthesis pathway was one of the top two obviously changed pathways, which may be active in both tuberous root initiation and the secondary thickening process in turnip. However, changes in the phenylpropanoid biosynthesis pathway during turnip taproot development remain unclear. Phenylpropanoid and isoquinoline alkaloid biosynthesis pathways share the same upstream substrate, tyrosine. Although the levels of several tyrosine metabolites decreased from S2T to S3T, multiple genes involved in the phenylpropanine pathway were downregulated. Changes in the phenylpropanine pathway were consistent with those observed in the root development of sweet potato (*Ipomoea batatas*). In sweet potatoes, the reduction in carbon flow toward phenylpropanoid biosynthesis and its delivery to carbohydrate metabolism and starch biosynthesis occurs at the earliest stage of storage root formation [21]. Therefore, we propose that the downregulated phenylpropanoid biosynthesis pathway triggers a tyrosine bias toward the upregulated (S)-coclaurine in the isoquinoline alkaloid synthesis pathway and may provide more substances for starch accumulation, which is beneficial for increased medicinal value and enlargement of the *S. kwangsiensis* bulbous stem.

Lignin synthesis is part of phenylpropanoid biosynthesis. COMT is the key enzyme involved in lignin biosynthesis. Lignin content decreased significantly in the *COMT* mutant [22–24]. Another key enzyme, peroxidase, catalyzes the final step of lignin biosynthesis [25]. Coniferyl alcohol is the precursor of guaiacyl lignin, or is further transformed into 5-Hydroxy-coniferylalcohol, which is the precursor of 5-hydroxygraiacyl lignin [26, 27]. Sinapaldehyde is a substrate that directly produces syringyl monolignols used for syringyl lignin biosynthesis [28]. Transcriptome analysis of sweet potato roots by Firon et al. [21] revealed downregulated lignin biosynthesis and upregulated starch biosynthesis in the early stages of storage root formation. Upregulated starch biosynthesis and downregulated lignin biosynthesis were also reported during the development of another traditional plant medicine, *Callerya speciosa* [29]. Angiosperm lignins are complex phenolic polymers that predominantly consist of guaiacyl and syringyl units with small amounts of p-hydroxyphenyl units. Monolignols are synthesized in the cytosol and transported to the cell wall, where they are oxidized to form lignins [30]. From a functional perspective, lignins impart strength to cell walls, facilitating rigidity and hydrophobicity [31, 32]. Togari [33] proposed a direct link between lignification and the initiation of storage roots in sweet potatoes, suggesting that lignification inhibits the development of storage roots. Liu et al. [14] proposed that GA induces DELLA protein degradation to release NAC proteins and induces xylem lignification, thereby inhibiting turnip taproot formation. In the bulbous stem of *S. kwangsiensis*, the expression of *COMT* and *peroxidase* was significantly higher in S1T than in S3T. In addition, coniferyl alcohol and sinapaldehyde, which are upstream substrates of lignin synthesis, were downregulated during *S. kwangsiensis* bulbous stem development. These results indicate the downregulation of the lignin synthesis pathway during *S. kwangsiensis* bulbous stem enlargement. We suggest that reduced carbon flow in the phenylpropanoid biosynthesis pathway may reduce the intermediate metabolites of lignin synthesis, along with the downregulation of key enzymes in the lignin biosynthesis pathway, leading to the overall downregulation of the lignin biosynthesis pathway, thus promoting the enlargement of storage bulbous stems. However, there were no significant changes in the transcription levels of *cinnamoyl coA reductase* (*CCR*) or *cinnamyl alcohol dehydrogenase* (*CAD*) during the development of *S. kwangsiensis* bulbous stems. These are key genes involved in lignin biosynthesis. CCR is the first rate-limiting enzyme that catalyzes the reaction of lignin-specific pathways, while CAD catalyzes the final step of monolignol biosynthesis [34, 35]. We speculate that the protein expression levels or activities of these two enzymes may change significantly during enlargement of the *S. kwangsiensis* bulbous stem. However, this hypothesis needs to be verified in further studies.

Plant hormones play important roles in plant growth and have been reported to be important in the initiation of taproot enlargement [12–14]. Multiple upregulated DAMs are involved in plant hormone signal transduction, and significant changes in plant signal transduction pathways at the transcriptional level occurred in S1T and S2T, but not in S2T and S3T, indicating that plant hormones are important for the initiation of the rapid expansion of bulbous stems in *S. kwangsiensis*. Plant hormones have been reported to regulate sugar metabolism and transport, including starch accumulation [23–27]. Sugars and plant hormones affect turnip taproot initiation and development by stimulating vascular cambium activity [14]. Therefore, carbohydrate metabolism is not only regulated by plant hormones but may also jointly affect the initiation of hypocotyl-originated bulbous stem enlargement with hormones. Plant hormones also play an important role in regulating the biosynthesis of alkaloids, including isoquinoline alkaloids [33]. However, the effects of plant hormones on carbohydrate metabolism and alkaloid synthesis pathways during *S. kwangsiensis* bulbous stem development require further investigation.

## Conclusion

We performed a detailed metabolome and transcriptome analysis at different time points during *S. kwangsiensis* bulbous stem development. Our results indicated that a large number of DAMs and DEGs are involved in bulbous stem development. A series of biological pathways were identified in which multiple significantly altered genes were enriched. These pathways included plant signal transduction, isoquinoline alkaloid biosynthesis, pentose and glucuronate interconversions, phenylpropanoid biosynthesis, and tyrosine metabolism. Based on this analysis, we propose that carbon tends to flow into alkaloid synthesis and storage of lipids and starch rather than into the downregulated phenylpropanoid biosynthesis pathway during the development of *S. kwangsiensis* bulbous stems. The decrease in metabolites involved in tyrosine metabolism may be one of the reasons for the downregulation of the phenylpropanoid biosynthesis pathway. Downregulation of lignin synthesis during phenylpropanoid biosynthesis may reduce the inhibition of bulbous stem growth, thereby directly promoting bulbous stem enlargement. The roles of different plant hormones in the rapid expansion of *S. kwangsiensis* bulbous stems require further study. These results provide practical guidance for breeding, cultivation, and harvesting of plants.

## Materials and methods

### Plant materials

The seeds used in the experiment were sourced from Hechi (Guangxi, China), and the plants were grown in a greenhouse. Seeds were sown in peat soil and vermiculite at a volume ratio of 1:1 and cultured at room temperature to keep the substrate moist. No nutrients or fertilizers were added prior to sampling. Seedlings were collected during three phenotypically diverse hypocotyl development periods. S1T: When the seedlings grew to a height of 5–10 cm after 20 d of seed germination and the base of the plant developed from the epicotyl had not expanded, we collected a portion of the seedlings approximately 2 cm above the ground. S2T: When the seedlings grew to a height of 50–80 cm after three months of seed germination, and the growth of the base of the plant developed from the epicotyl entered a period of rapid expansion, we collected the expanding part approximately 2 cm above the ground. S3T: Bulbous stems were selected from wild plants that were healthy, free from pests and diseases, and had been growing for five years. Bulbous stem centers were collected for the experiments. The sampling process was conducted in a sterile environment, and the samples were quickly frozen in liquid nitrogen and stored at –80 °C.

### Paraffin sections

The specimens were fixed in FAA solution (formalin (37%): glacial acetic acid: ethanol (50%), ratio 5:5:90 in volume) [36]. Samples were progressively dehydrated in a graded ethanol series (70–100%), embedded in paraplasts, and mounted on block holders. Samples were sectioned in 8-μm slices using a Reichert 820H Histostat rotary microtome (Warer-Lambert Tech. Inc., USA). The paraffin sections were affixed to slides, stained with a combination of safranin and fast green, covered with a cover slip in place with a thin coating of Neutral Balsam, and dried at 38 °C for 48 h [36]. All the sections were observed and photographed using a Leica DMLB microscope (Leica Microsystems, Germany).

### Metabolite extraction and UPLC-MS/MS analysis

The metabolite extracts were freeze-dried under vacuum and ground to a powder (30 Hz, 1.5 min). Powdered plant tissues (50 mg) were extracted using 1.2 mL precooled 70% methanol. Vortex oscillation was conducted every 30 min for 30 s, six times. The solutions were centrifuged at 12,000 rpm for 3 min before the supernatant was transferred to a new 1.5-mL Eppendorf tube. The insoluble fraction was filtered using a microporous membrane (0.22 μm) and stored in a sample vial for UPLC-MS/MS analysis.

UPLC was performed using a SHIMADZU Nexera X2 and Tandem mass spectrometry (MS/MS) analysis was conducted using an Applied Biosystems 6500 QTRAP with an Agilent SB-C18 1.8 µm, 2.1 × 100 mm column. The injection volume was 2 µL and a binary separation gradient was applied at a flow rate of 0.35 mL/min: 0 min, isocratic 95% A (ultra-pure water with 0.1% formic acid), 5% B (acetonitrile with 0.1% formic acid); 0 to 9 min, linear gradient to 95% B; 9 to 10 min, isocratic 95% B; 10 to 11.1 min, linear gradient to 5% B. The main conditions of mass spectrometry were electrospray ionization (ESI) source temperature 500 °C; ion spray voltage (IS) 5500 V (positive ion mode) /-4500 V (negative ion mode); ion-source gas I (GSI), gas II (GSII), and curtain gas (CUR) were set to 50, 60, and 25 psi, respectively. Collision-induced ionization parameters were set to “high.” The metabolites were quantified via multiple reaction monitoring (MRM) analysis using triple quadrupole mass spectrometry based on a self-established software database (MWDB) [37, 38].

### Metabolome analysis

Metabolomic analysis was performed using the MetaboAnalystR (1.0.1) package in R. To identify DAMs, we implemented orthogonal partial least squares discriminant analysis (OPLS-DA) using MetaboAnalystR, according to the following thresholds: variable importance in projection (VIP) score ≥ 1 and absolute log2 FC ≥ 1. Pathway enrichment analysis of the identified metabolites was performed by mapping them to the Kyoto Encyclopedia of Genes and Genomes database. The significant pathways of the DAMs were determined using the *P*-values obtained from the hypergeometric test. PCA was performed using the statistical function prcomp in the R platform.

### RNA extraction and RNA-Seq

Total RNA was extracted from *S. kwangsiensi* seedlings from three biological replicates at each stage using a Qiagen RNeasy Plant Kit (Hilden, Germany) according to the manufacturer’s protocol. DNA contamination and the quality, concentration, and integrity of the total RNA were confirmed using agarose gel electrophoresis, NanoPhotometer, Qubit 2.0 fluorometer, and Agilent 2100 BioAnalyzer.

*S. kwangsiensi* seedling RNA-seq libraries were prepared using the Illumina TruSeq RNA Sample Prep Kit, following the manufacturer’s instructions, and the quality of the library was detected using Qubit2.0 and Q-PCR. The cDNA library products that passed quality tests were sequenced using the Illumina HiSeq-2500 platform.

### Transcriptome analysis

To obtain high-quality clean reads, the read sets obtained from *S. kwangsiensi* seedlings were subjected to adapter removal and quality analysis using CASAVA (1.8.2, Illumina). Read sets with N content exceeding 10% of the number of read bases were considered low-quality sequences and were filtered out. The Trinity software package was used for efficient and robust de novo assembly of clean reads. All read pairs from *S. kwangsiensi* retained after filtering were used for de novo transcriptome assembly using Trinity (version 2.6.6) with default parameters to construct unigenes.

The unigenes were functionally annotated and classified using various databases, including nr protein, Swiss-Prot, KEGG, TREMBL, Gene Ontology (GO), and Clusters of Orthologous Groups of Proteins (COG) using BLAST software. First, we selected the NR, Swiss-Prot, KEGG, and COG databases to confirm sequence directions. Alignment of the unigene and protein databases was performed using BLASTx. Finally, the protein sequences of unigenes with the highest similarity were retrieved for functional annotation and classification.

Clean reads from *S. kwangsiensi* were aligned to assemble the transcripts using Botwie2. Gene expression was calculated using RNA-seq by Expectation Maximization (RSEM). The expression value of each unigene was normalized to fragments per kilobase of transcript per million fragment-mapped reads (FPKM). To identify differentially expressed genes, we used the DEseq2 package (1.22.2) in R to analyze unstandardized read count data between two samples based on a false discovery rate (FDR) < 0.05, and absolute log2 FC ≥ 1.

### Temporal analysis

The short time-series expression miner (STEM) software can process short time-series data for clustering and statistical biological explanations using exclusive approaches and integrate them with the GO and KEGG databases. We used the STEM algorithm with default parameters to analyze changes in the gene expression profiles of *S. kwangsiensi* during development. The DEGs of *S. kwangsiensi* were clustered according to their *P*-values. Clustered profiles with *P* ≤ 0.05 were considered differentially expressed. Genes within the selected clusters were enriched in GO terms and KEGG pathways for functional annotation, using a hypergeometric distribution test. Functional items of each selected cluster with Q-values ≤ 0.05 were retained.

### qRT-PCR analysis

Quantitative reverse transcription PCR (qRT-PCR) was performed to validate the expression of the key genes. We extracted RNA from the bulbous stems at the three developmental stages and converted it into cDNA using a PrimeScript Reverse Transcriptase kit (Takara). Specific primers of key genes for qRT-PCR were designed using Primer Premier software (version 5.0) (Table S6). qRT-PCR was performed using a qTOWER 3G Real-Time PCR Detection System. All reactions were performed in triplicates. We used 25S RNA as an internal control for gene expression normalization and the 2 ^(− ΔΔCt)^ algorithm to estimate gene expression values [39].

### Statistical analysis

Three replicates were analyzed for each tissue type at each stage. Pearson’s correlation coefficients were calculated between the abundance of different genes and proteins from metabolomic profiling and between the relative expression from qRT-PCR and RNA-seq across stages using R v3.6.3.

### Supplementary Information

**Supplementary Material 1.**

**Supplementary Material 2.**

## Data Availability

The datasets used and/or analysed during the current study are available in the NCBI Bioproject repository, [PRJNA980934].

## Abbreviations

UPLC-MS/MS: Ultraperformance liquid chromatography tandem mass spectrometry
DAMs: Differential accumulation of metabolites
DEGs: Differential expressed genes
PCA: Principal component analysis
VIP: Variable importance in projection
KEGG: Kyoto Encyclopedia of Genes and Genomes
COG: Clusters of Orthologous Groups
KOG: EuKaryotic Orthologous Groups
NR: NCBI non-redundant
ABA: Abscisic acid
OPLS-DA: Orthogonal partial least squares discriminant analysis
RSEM: RNA-seq by Expectation Maximization
FPKM: Fragments per kilobase of transcript per million fragments mapped reads
FDR: False discovery rate
STEM: Short time-series expression miner
GO: Gene Ontology
