# Supplementary material for: Integrated metabolomic and transcriptomic analyses provide insights into regulation mechanisms during bulbous stem development in the Chinese medicinal herb plant, Stephania kwangsiensis
Source: BMC Plant Biol. 2024 Apr 11;24:276. doi: 10.1186/s12870-024-04956-2 (PMC11007893; doi:10.1186/s12870-024-04956-2)
Supplement: Supplementary file 1 — Supplementary Material 1. [file 12870_2024_4956_MOESM1_ESM.pptx]

## Slide 1
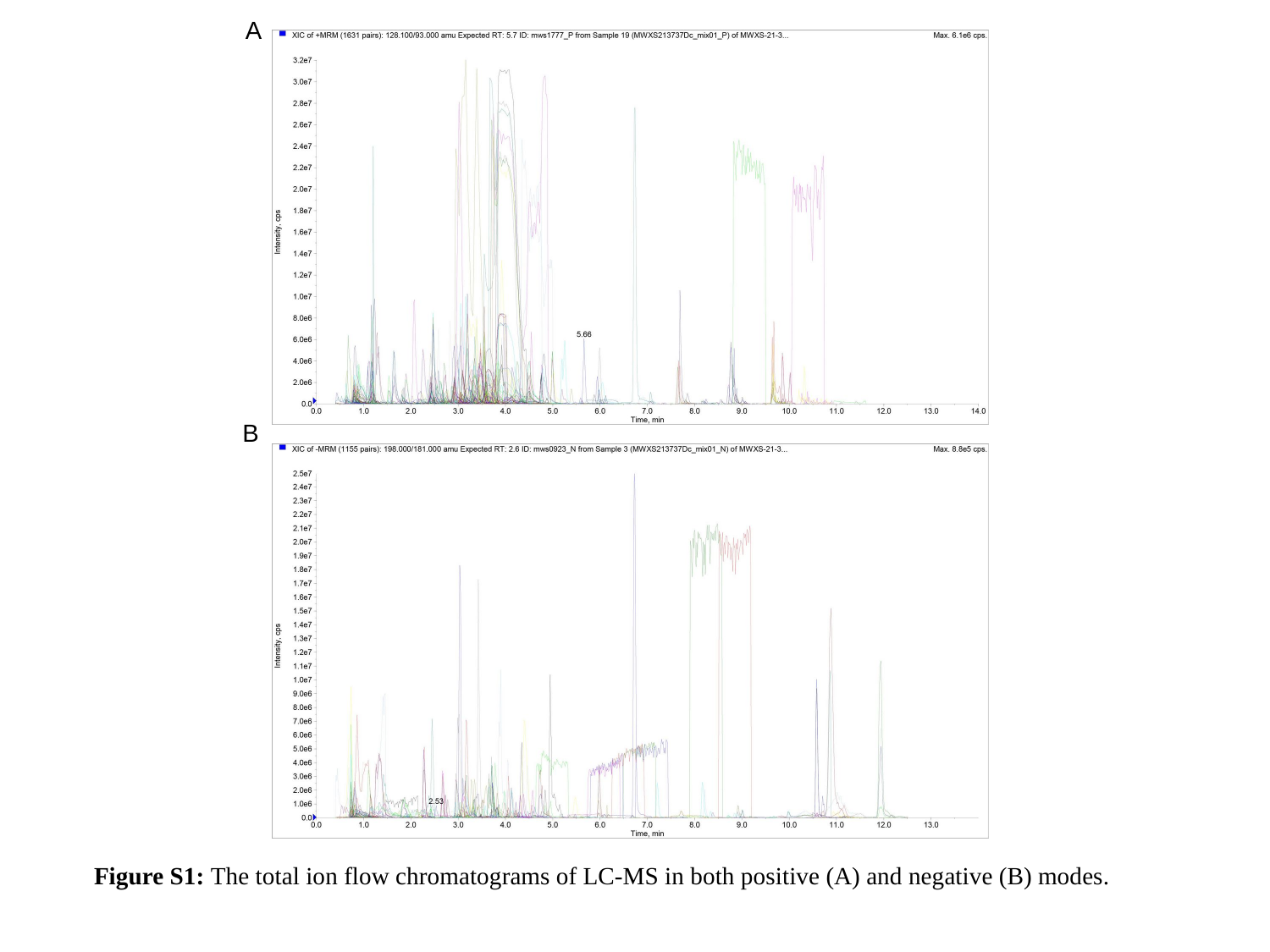

A
B
Figure S1: The total ion flow chromatograms of LC-MS in both positive (A) and negative (B) modes.

## Slide 2
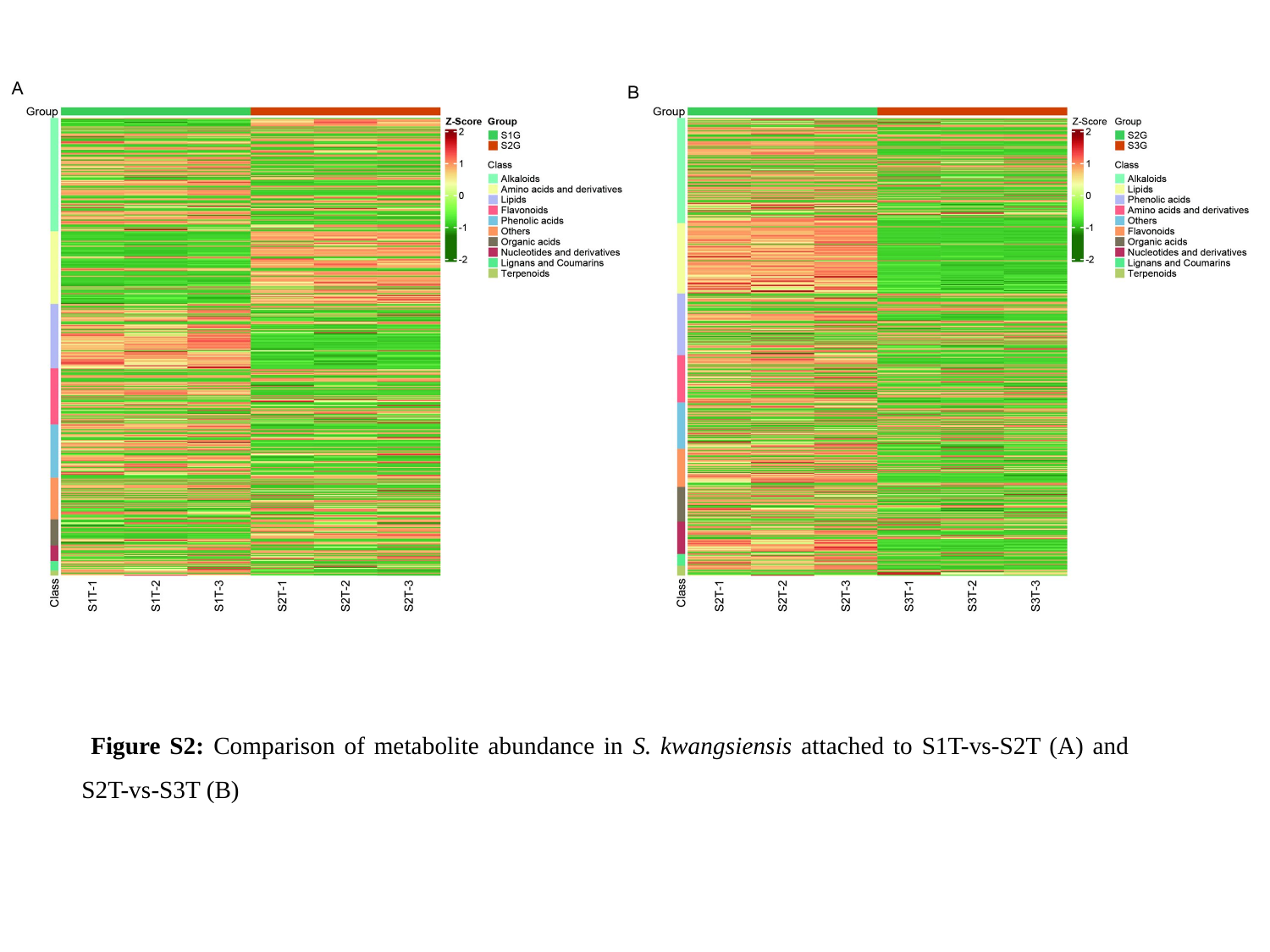

Figure S2: Comparison of metabolite abundance in S. kwangsiensis attached to S1T-vs-S2T (A) and S2T-vs-S3T (B)

## Slide 3
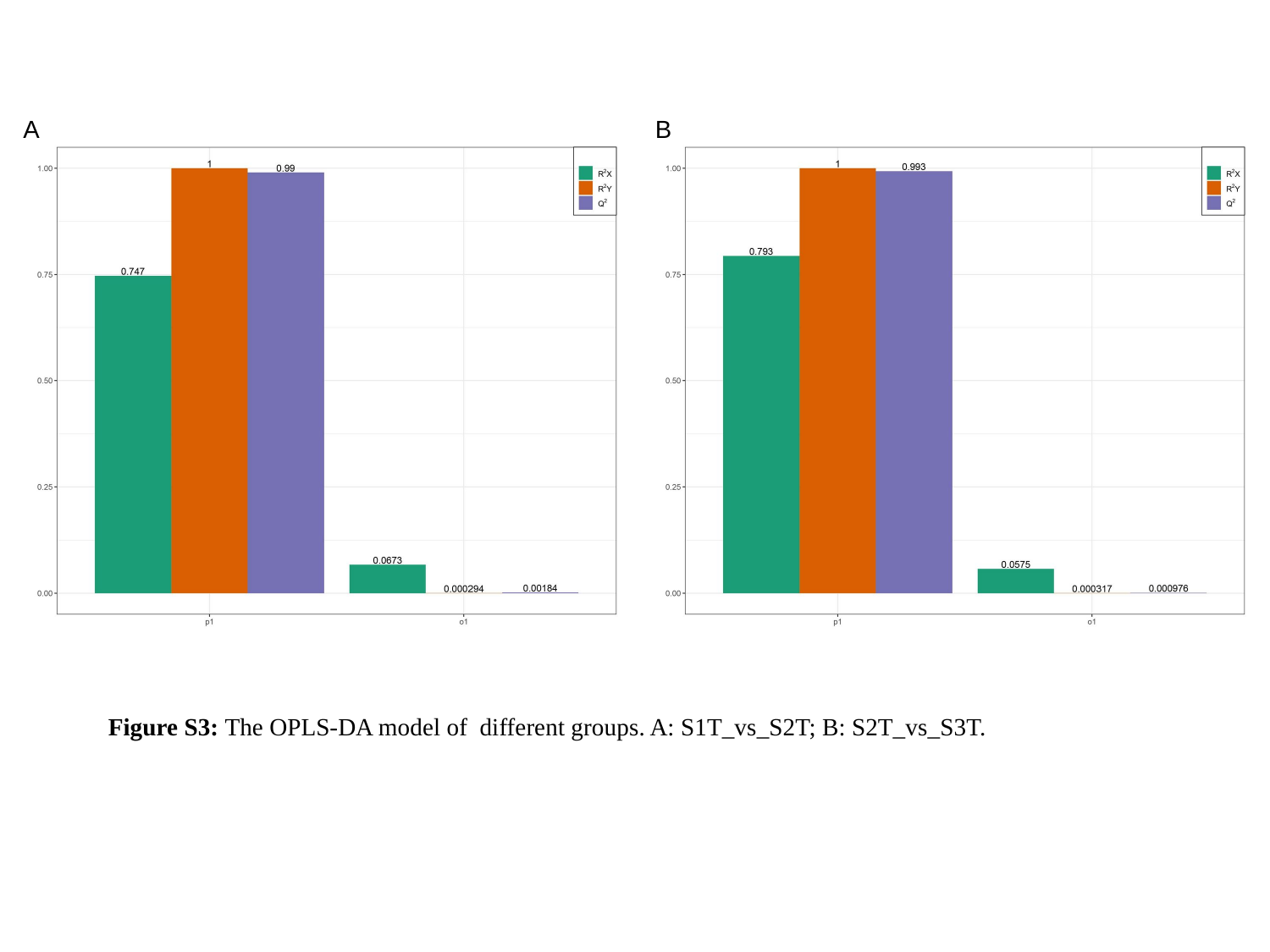

A
B
Figure S3: The OPLS-DA model of different groups. A: S1T_vs_S2T; B: S2T_vs_S3T.

## Slide 4
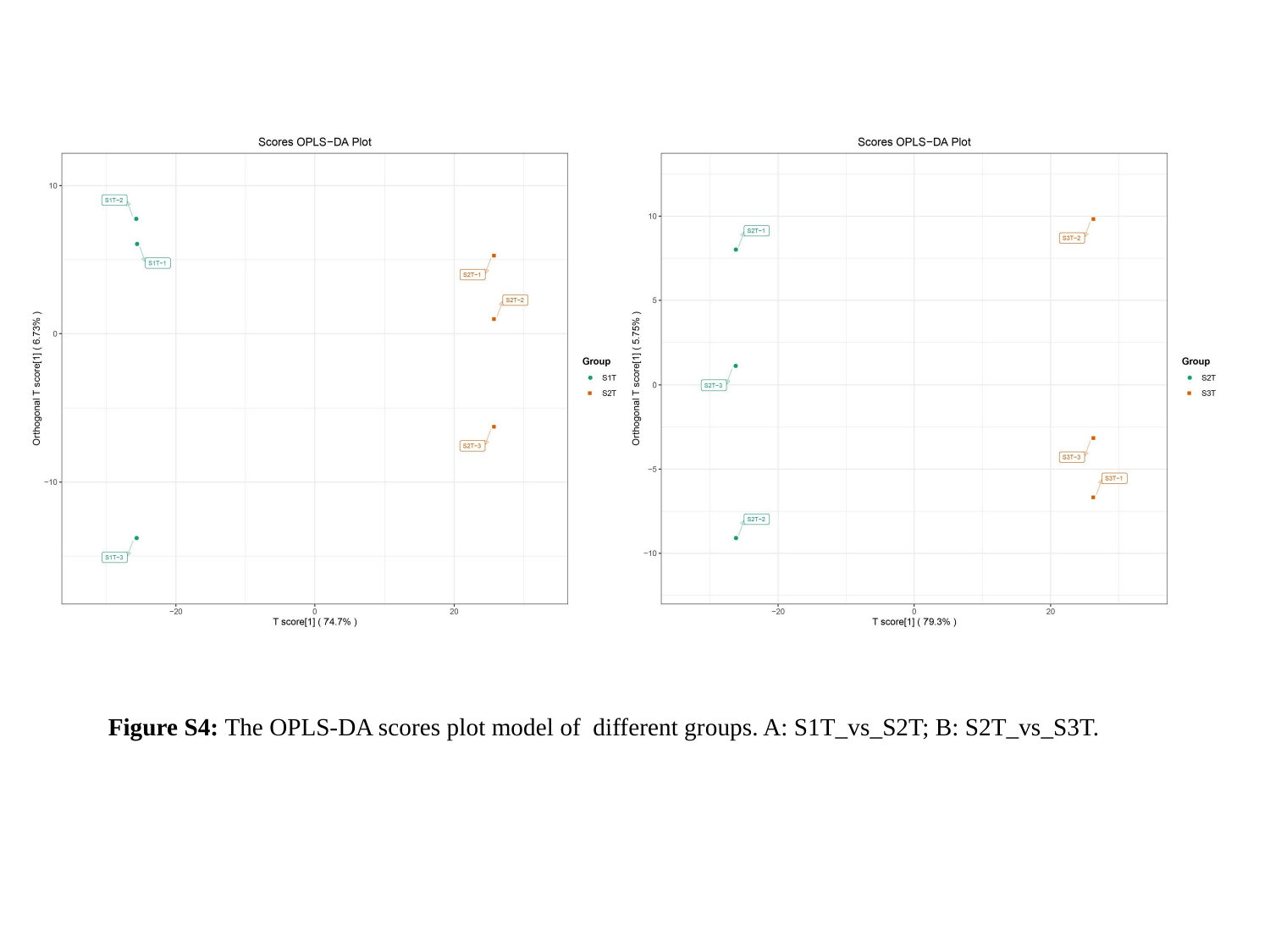

Figure S4: The OPLS-DA scores plot model of different groups. A: S1T_vs_S2T; B: S2T_vs_S3T.

## Slide 5
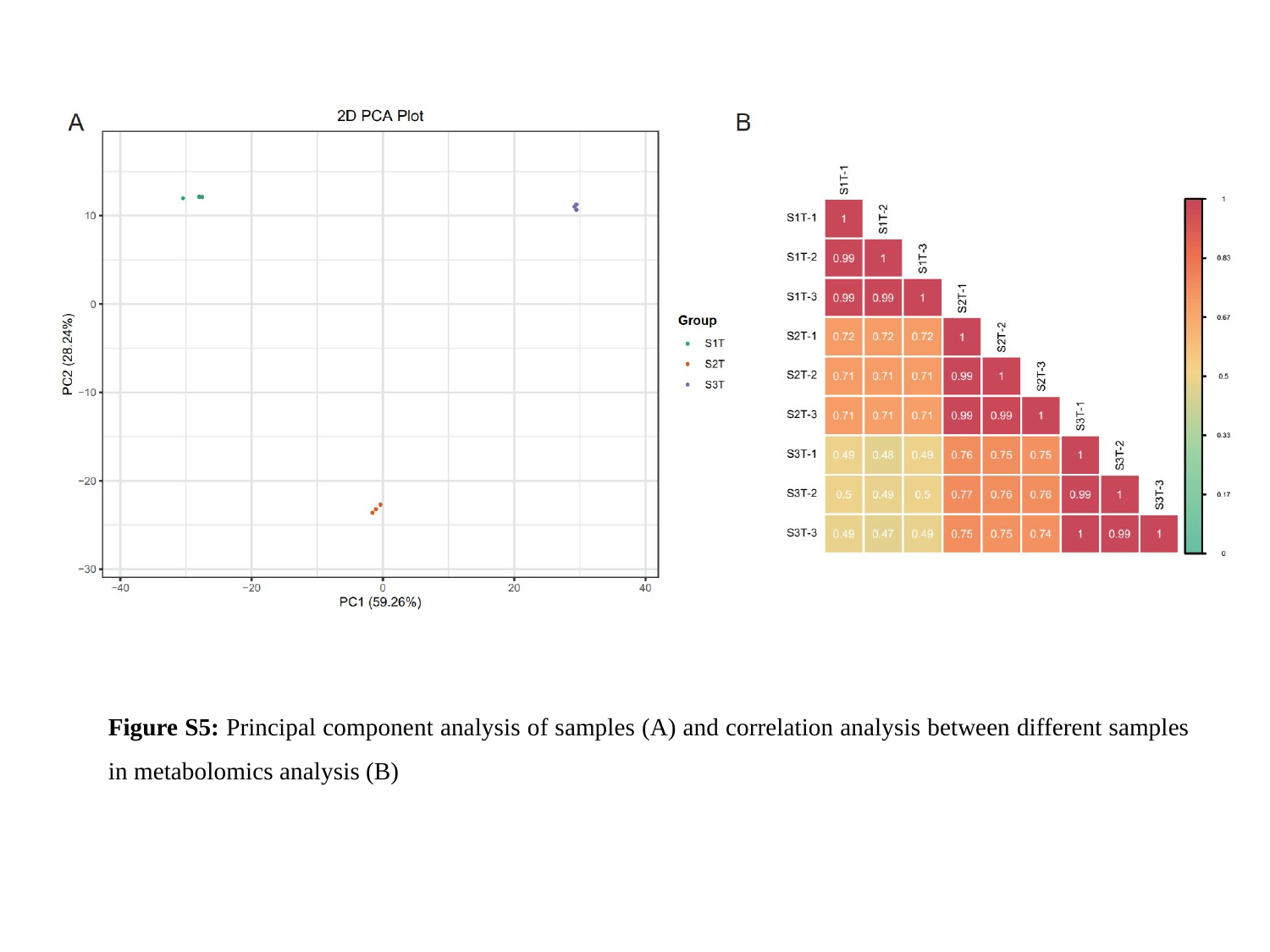

Figure S5: Principal component analysis of samples (A) and correlation analysis between different samples in metabolomics analysis (B)

## Slide 6
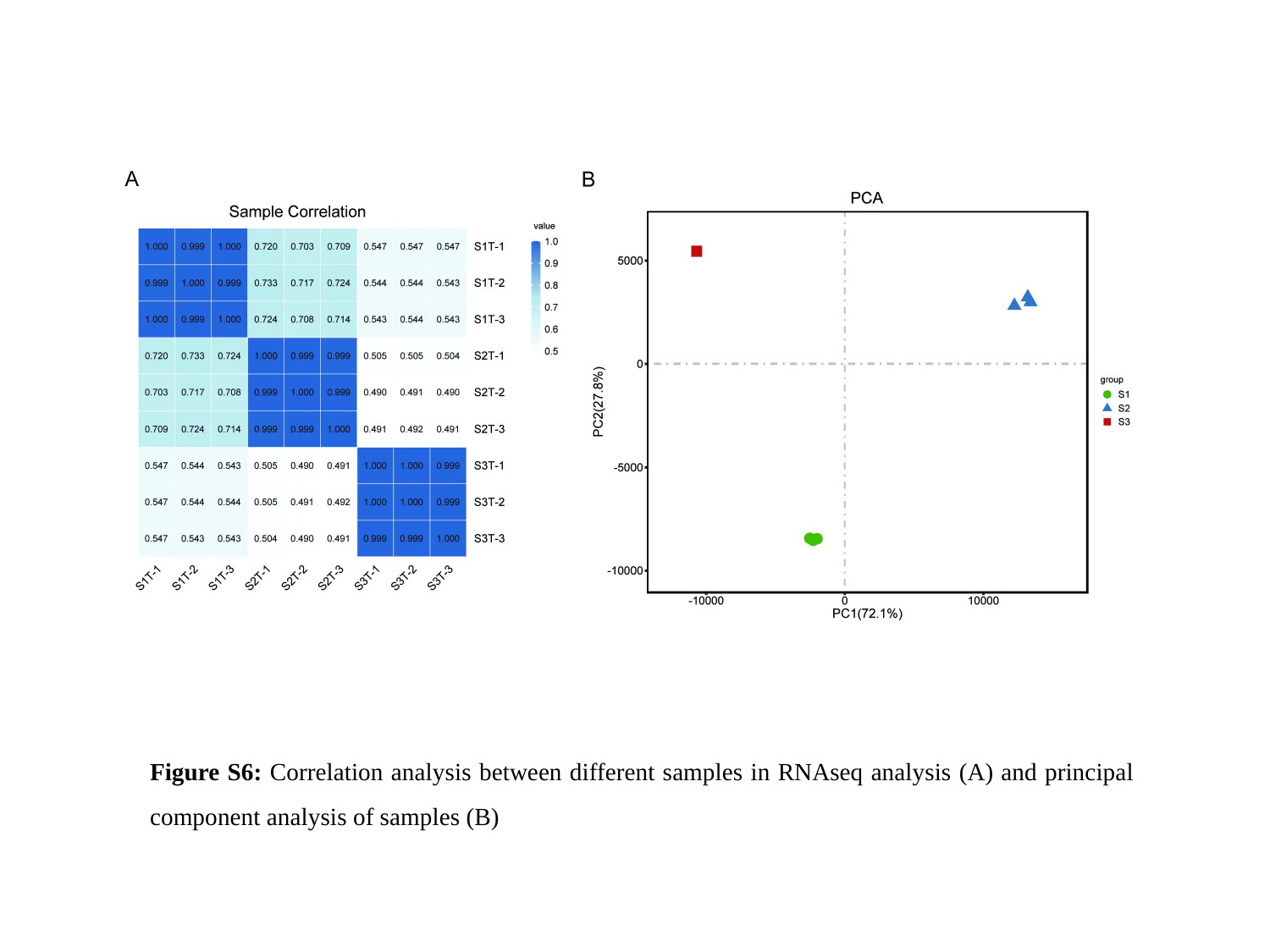

Figure S6: Correlation analysis between different samples in RNAseq analysis (A) and principal component analysis of samples (B)

## Slide 7
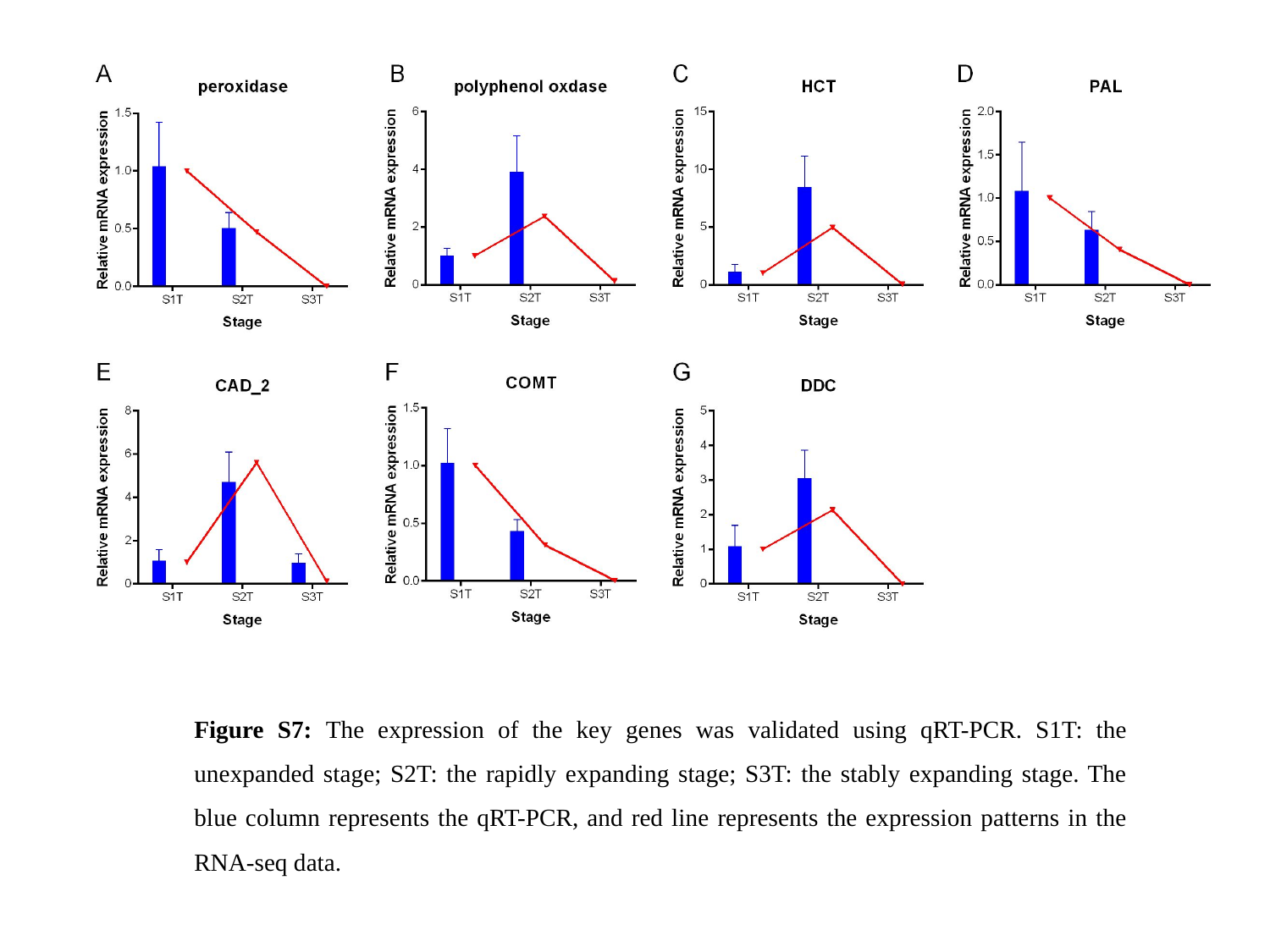

Figure S7: The expression of the key genes was validated using qRT-PCR. S1T: the unexpanded stage; S2T: the rapidly expanding stage; S3T: the stably expanding stage. The blue column represents the qRT-PCR, and red line represents the expression patterns in the RNA-seq data.

## Slide 8
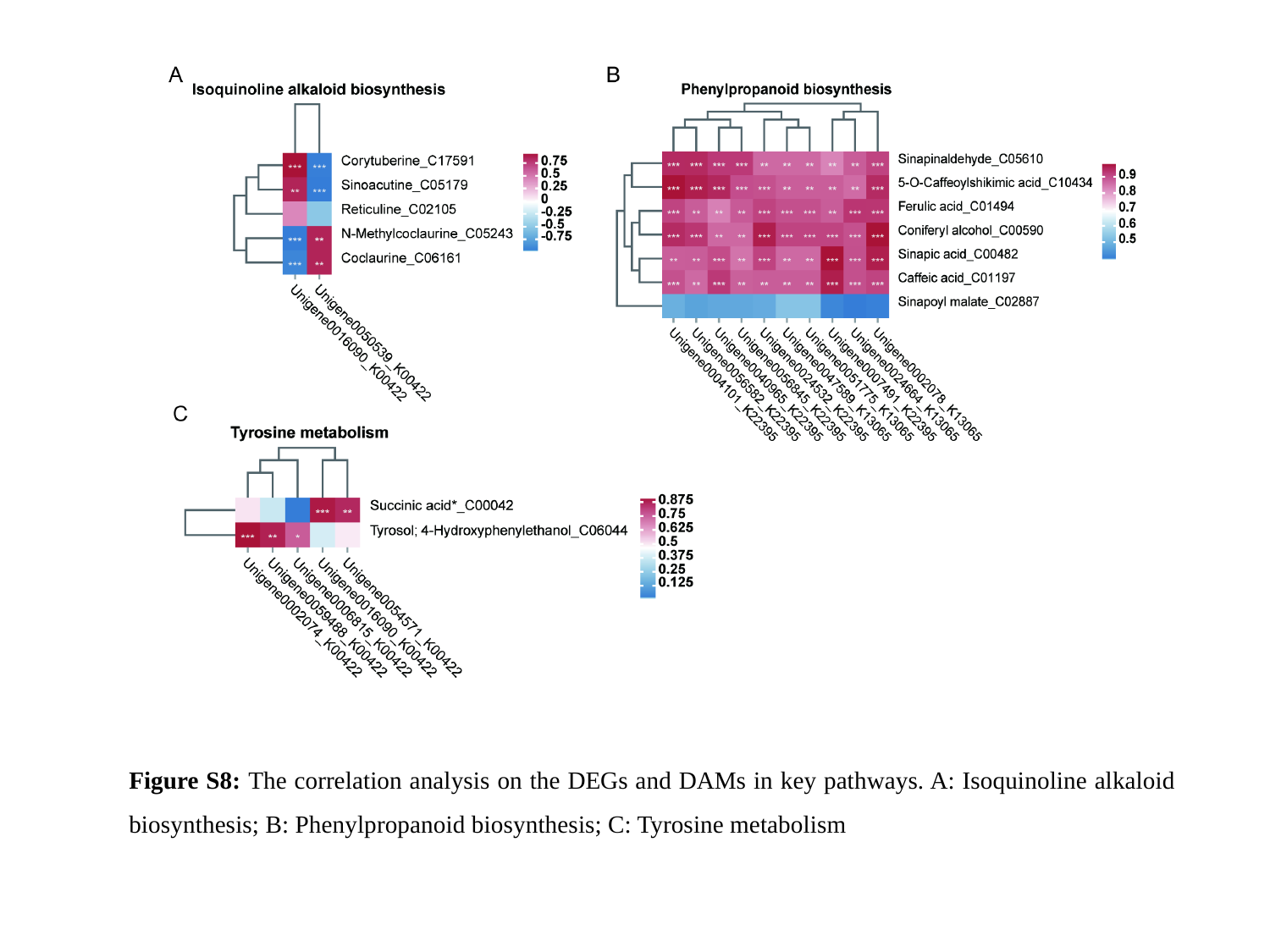

Figure S8: The correlation analysis on the DEGs and DAMs in key pathways. A: Isoquinoline alkaloid biosynthesis; B: Phenylpropanoid biosynthesis; C: Tyrosine metabolism
